# Supplementary material for: Harnessing CRISPR interference to resensitize laboratory strains and clinical isolates to last resort antibiotics
Source: Sci Rep. 2025 Jan 2;15:261. doi: 10.1038/s41598-024-81989-5 (PMC11696610; doi:10.1038/s41598-024-81989-5)
Supplement: Supplementary file 1 — Supplementary Material 1 [file 41598_2024_81989_MOESM1_ESM.pdf]

Supplementary Information for

Harnessing CRISPR interference to resensitize laboratory strains and clinical isolates to last resort antibiotics

Angelica Frusteri Chiacchiera[1,2,3], Michela Casanova[1,2], Massimo Bellato[4], Aurora Piazza[5,6], Roberta Migliavacca[5,6], Gregory Batt[3], Paolo Magni[1,2,*], Lorenzo Pasotti[1,2,3,*,†]

[1]Department of Electrical, Computer and Biomedical Engineering, University of Pavia, Via Ferrata 5, Pavia, Italy.
[2]Centre for Health Technologies, University of Pavia, Via Ferrata 5, Pavia, Italy.
[3]Institut Pasteur, Inria, Université Paris Cité, 28 rue du Docteur Roux, Paris, France.
[4]Department of Information Engineering, University of Padua, Via Gradenigo 6b, 35131 Padua, Italy.
[5]Department of Clinical-Surgical, Diagnostic and Pediatric Sciences, University of Pavia, Viale Brambilla 74, Pavia, Italy.
[6]Fondazione IRCCS Policlinico San Matteo, Pavia, Italy.
[*]These authors have contributed equally to this work and share last authorship.
[†]Correspondence: Lorenzo Pasotti - lorenzo.pasotti@unipv.it.

Content

Figure S1. 2
Figure S2. 3
Figure S3. 3
Figure S4. 4
Figure S5. 4
Figure S6. 5
Figure S7. 6
Figure S8. 7
Figure S9. 8
Figure S10. 9
Figure S11. 9
Figure S12. 10
Figure S13. 11
Figure S14. 12
Figure S15. 12
Figure S16. 13
Figure S17. 13
Figure S18. 14
Figure S19. 15
Figure S20. 16
Table S1....................17
Table S2....................24
Table S3....................26
Table S4....................26
Table S5....................26
Table S6....................28

Supplementary Figures

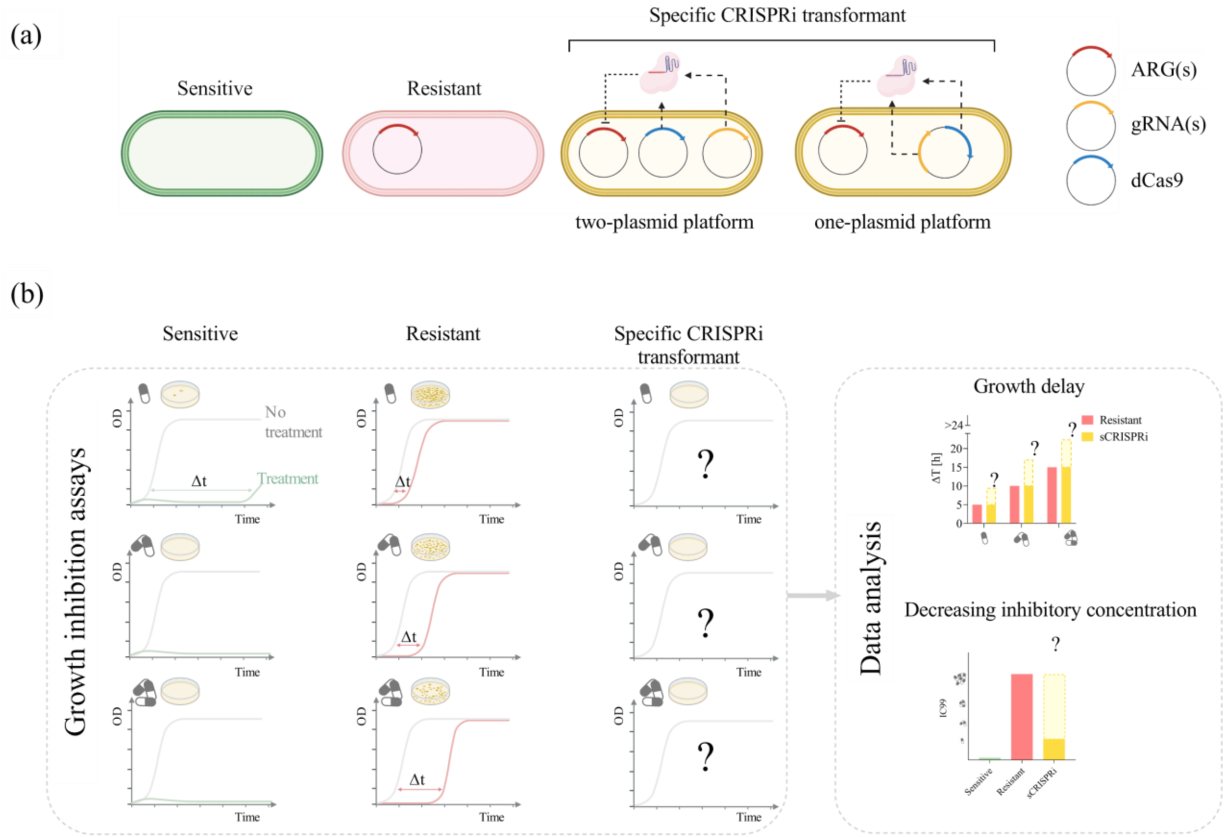

Figure S1.

Characterization methods for quantifying the CRISPRi-mediated re-sensitization to target antibiotics. (a) Description of the test strains: *sensitive*, lacking the ARG; *resistant*, bearing the ARG; *specific CRISPRi transformant* (*sCRISPRi*), bearing the ARG and the CRISPRi platform. Plasmids with their main components: ARG (red), dCas9 module (blue); and sgRNA module (yellow). The ARG(s) can be located on one plasmid, two plasmids or on the chromosome, depending on the considered case study. The dCas9 module can be driven by a weak constitutive promoter in a medium copy plasmid or by an HSL-inducible promoter in a low copy plasmid. The gRNA module has been implemented to transcribe one or two sgRNAs under an IPTG-inducible promoter in a low-copy plasmid. A constitutive dCas9 module and an IPTG-inducible sgRNA module constitute a two-plasmid CRISPRi platform. An HSL-inducible dCas9 module and an IPTG-inducible sgRNA module constitute a one-plasmid CRISPRi platform**.** **(b)** CRISPRi-mediated re-sensitization is quantified via growth inhibition assays. Different concentrations of target antibiotics are added in liquid media to perform microplate assays (on the left), or solid media, to perform agar plate assays (on the right). The expected phenotypes are illustrated as a growth profile in response to antibiotic treatment (illustrated as pills) with the relative growth delays from microplate assays, and IC_{99} from agar plate assays.

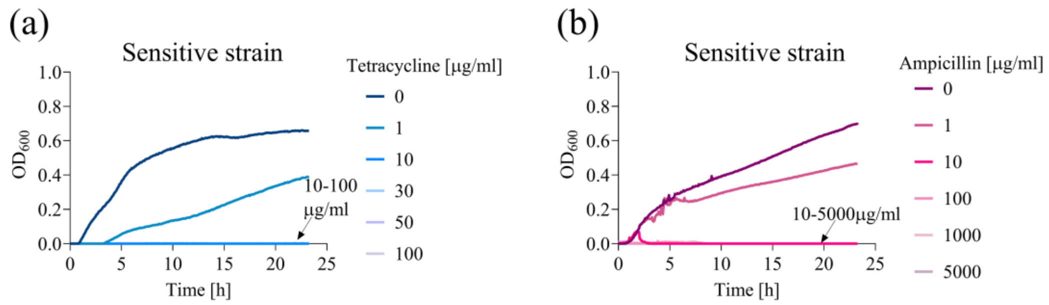

Figure S2.

Growth curves of the *sensitive* strains in the presence of tetracycline or ampicillin. (a-b) Representative curves are shown from a set of at least three independent experiments with *sensitive* strains treated with tetracycline (a) or ampicillin (b). Data come from experiments in LB media with IPTG. TOP10 and TOP10F' were used as *sensitive* strains for tetracycline and ampicillin, respectively.

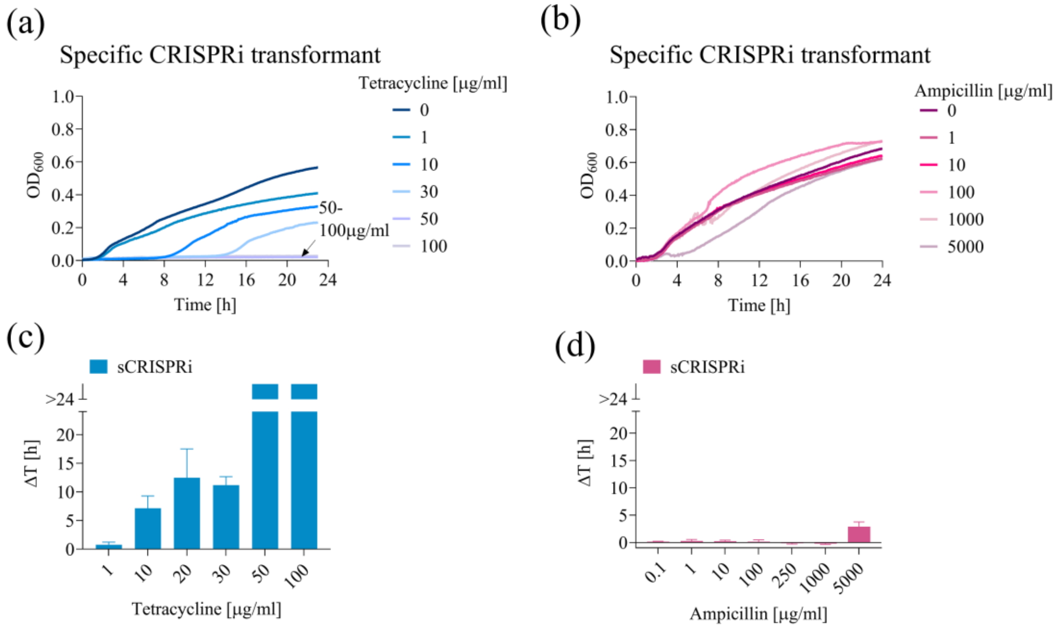

Figure S3.

Re-sensitization of laboratory strains to tetracycline and ampicillin by single ARG targeting, in the absence of IPTG. (a-b) Growth profiles of *sCRISPRi* strains from microplate assays to investigate tetracycline (a) and ampicillin (b) re-sensitization. *sCRISPRi* strains are engineered with a CRISPRi platform, including constitutive dCas9 and IPTG-inducible gtetA or $gbla_{promoter}$ targeting *tetA* or $bla_{TEM-116}$, respectively. The overlapped curves in which a complete growth inhibition was achieved are indicated with an arrow, with the list of the corresponding antibiotic concentrations. Representative curves are shown from a set of at least three independent experiments. **(c-d)** Growth delays (Δt) of treated strains relative to the growth profile without antibiotics for the *resistant* and *sCRISPRi* strains. Bars represent means and standard deviations (N=3). The bars over the interrupted axis indicate the antibiotic concentration (MIC) for which OD_{600} was lower than 0.1. Different from Figure 1 in the main text, all the data presented here have been measured without IPTG that triggers sgRNA expression. $T\text{-}cr_{sgT\_const}$ and $A\text{-}cr_{sgA\_const}$ were used as *sCRISPRi* strains to investigate tetracycline and ampicillin re-sensitization, respectively.

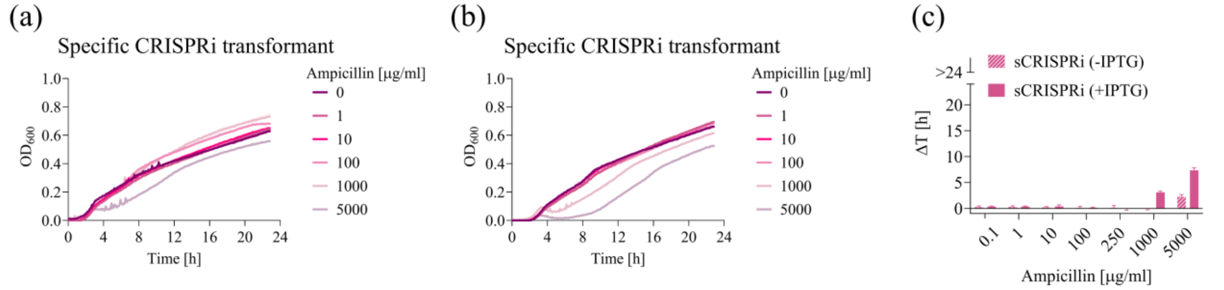

Figure S4.

Re-sensitization of laboratory strains to ampicillin by targeting the *bla*$_{TEM-116}$ coding sequence, in the absence or presence of IPTG. (a-b) Growth profiles of *sCRISPRi* strain from microplate assays to investigate ampicillin re-sensitization. *sCRISPRi* strain is engineered with a two-plasmid CRISPRi platform, including constitutive dCas9 and IPTG-inducible gbla$_{CDS}$ targeting *bla*$_{TEM-116}$ CDS. Antibiotic concentrations are provided in the legend. Representative curves are shown from a set of at least three independent experiments. Growth profiles are shown without IPTG (a) and with IPTG (b) that induced gbla$_{CDS}$ expression. **(c)** Growth delays (Δt) of treated strains relative to the growth profile without antibiotics for the *resistant* and *sCRISPRi* strains, with/without IPTG as indicated in the legend. Bars represent means and standard deviations (N=3). The bars over the interrupted axis indicate the antibiotic concentration (MIC) for which OD_{600} was lower than 0.1. All the data come from experiments in LB media. A-cr$_{sgA(CDS)_const}$ was used as *sCRISPRi* strain to investigate ampicillin re-sensitization.

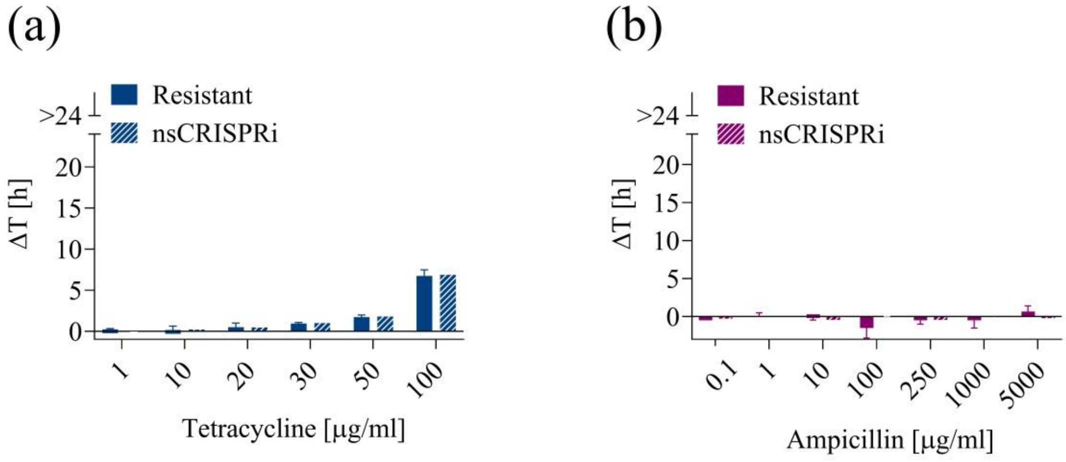

Figure S5.

Comparison of growth delays between resistant laboratory strain and resistant laboratory strain bearing a non-specific guide RNA. (a-b) Growth delays (Δt) of tetracycline (a) and ampicillin (b) treated strains relative to the growth profile without antibiotics for the *resistant* and *non-specific CRISPRi transformant* (*nsCRISPRi*) strains. Bars represent means and standard deviations (N=3). All the data come from experiments in LB media in the presence of IPTG. TOP10F' and T-cr$_{sgA_const}$ were used as *resistant* and *nsCRISPRi* strains to investigate tetracycline re-sensitization, respectively. A-res and A-cr$_{sgT_const}$ were used as *resistant* and *nsCRISPRi* strains to investigate ampicillin re-sensitization, respectively.

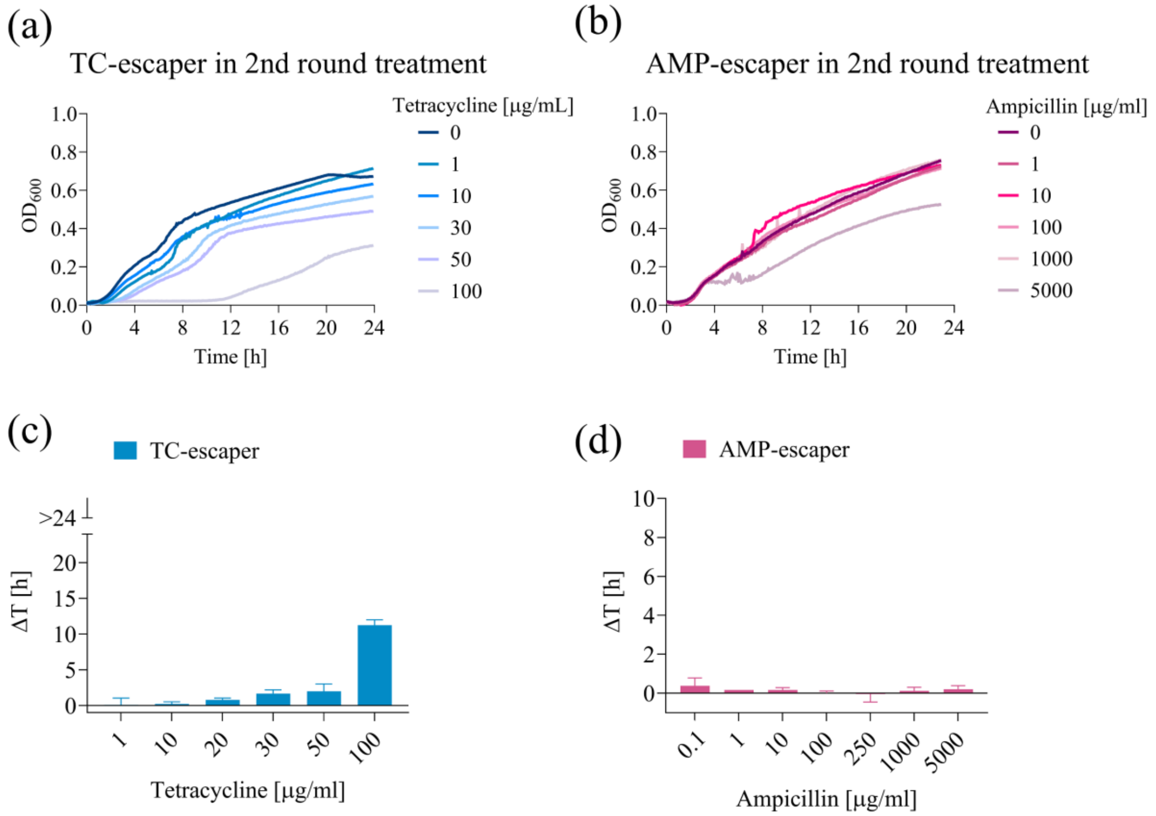

Figure S6.

Growth profiles of escaper mutants isolated from tetracycline and ampicillin microplate assays with laboratory strains. (a-b) Single colonies of sCRISPRi strains bearing gtetA or $gbla_{promoter}$, recovered from the highest AMP and TC concentration in microplate assays, were collected and used to carry out a second round of treatment with the same range of antibiotic concentrations as in the first round of treatment. Representative curves are shown from a set of at least three independent colonies. **(c-d)** Growth delays (Δt) are shown for the same strains relative to the growth profile without antibiotics. Bars represent means and standard deviations (N=3). Data come from experiments in LB media in the presence of IPTG. All the tested colonies exhibited a delay pattern comparable with the resistant strain, as evidenced by the decreased recovery time and the increased MIC values, suggesting that the isolated escapers were represented by mutants.

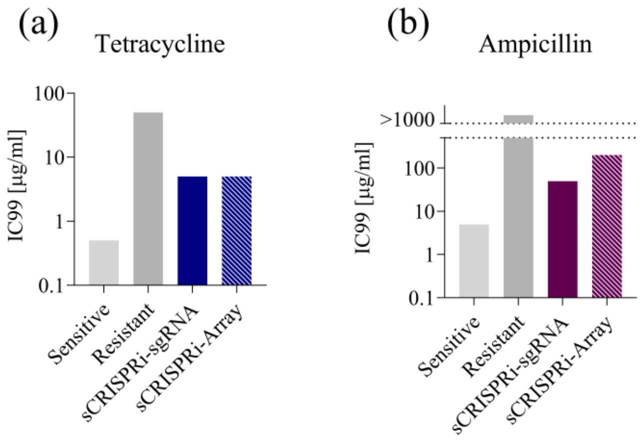

Figure S7.

Agar plate assays for the characterization of CRISPRi-mediated ARG inhibition.

(a-b) Antibiotic concentrations inhibiting >99% bacterial growth (IC_{99}) in agar plate assays for tetracycline (a) and ampicillin (b) treated strains. sCRISPRi-sgRNA refers to strains engineered with a two-plasmid CRISPRi platform, including a constitutive dCas9 module and an IPTG-inducible single gRNA module. sCRISPRi-Array refers to strains engineered with a one-plasmid CRISPRi platform, including a HSL-inducible dCas9 module and an IPTG-inducible gRNA module implemented as CRISPRi array. Bars represent IC_{99} recorded for two replicates that provided consistent values, therefore no error bars are present. Data come from experiments in LB media with IPTG or IPTG + HSL=10nM. TOP10, TOP10F', and T-cr_{sgT_const} and AT-cr_{arAT} were used as *sensitive*, *resistant*, and *sCRISPRi-sgRNA* and *sCRISPRi-Array* strains, respectively, to investigate tetracycline re-sensitization. TOP10F', A-res, and A-cr_{sgA_const} and AT-cr_{arAT} were used as *sensitive*, *resistant*, and *sCRISPRi-sgRNA* and *sCRISPRi-Array* strains, respectively, to investigate ampicillin re-sensitization.

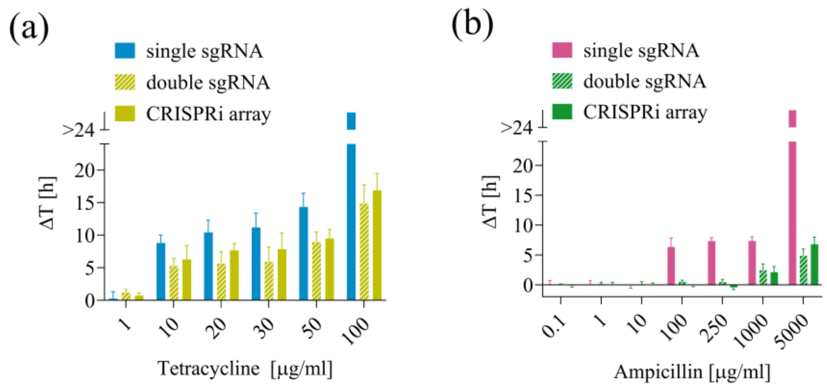

Figure S8.

Comparison of growth delays among single-sgRNA, double-sgRNA, and CRISPRi array designs, with a weak constitutive dCas9 expression cassette in laboratory strains. (a-b) Growth delays (Δt) of tetracycline (a) and ampicillin (b) *sCRISPRi* strains are shown for the indicated strains relative to the growth profile without antibiotics. *sCRISPRi* strains are engineered with a two-plasmid CRISPRi platform, including constitutive dCas9 and IPTG-inducible single sgRNA cassette, double-sgRNA cassette or CRISPRi array. Bars represent means and standard deviations (N=3). The bars over the interrupted axis indicate the antibiotic concentration (MIC) for which OD_{600} was lower than 0.1. All the data come from experiments in LB media in the presence of IPTG. T-cr_{sgT_const}, A-cr_{sgA_const} were used as *sCRISPRi* strains with single-sgRNA to investigate tetracycline and ampicillin re-sensitization, respectively. AT-cr_{sgAT_const} and AT-cr_{arAT_const} were used as *sCRISPRi* strains with double-sgRNA and CRISPRi array, respectively, to investigate tetracycline and ampicillin re-sensitization.

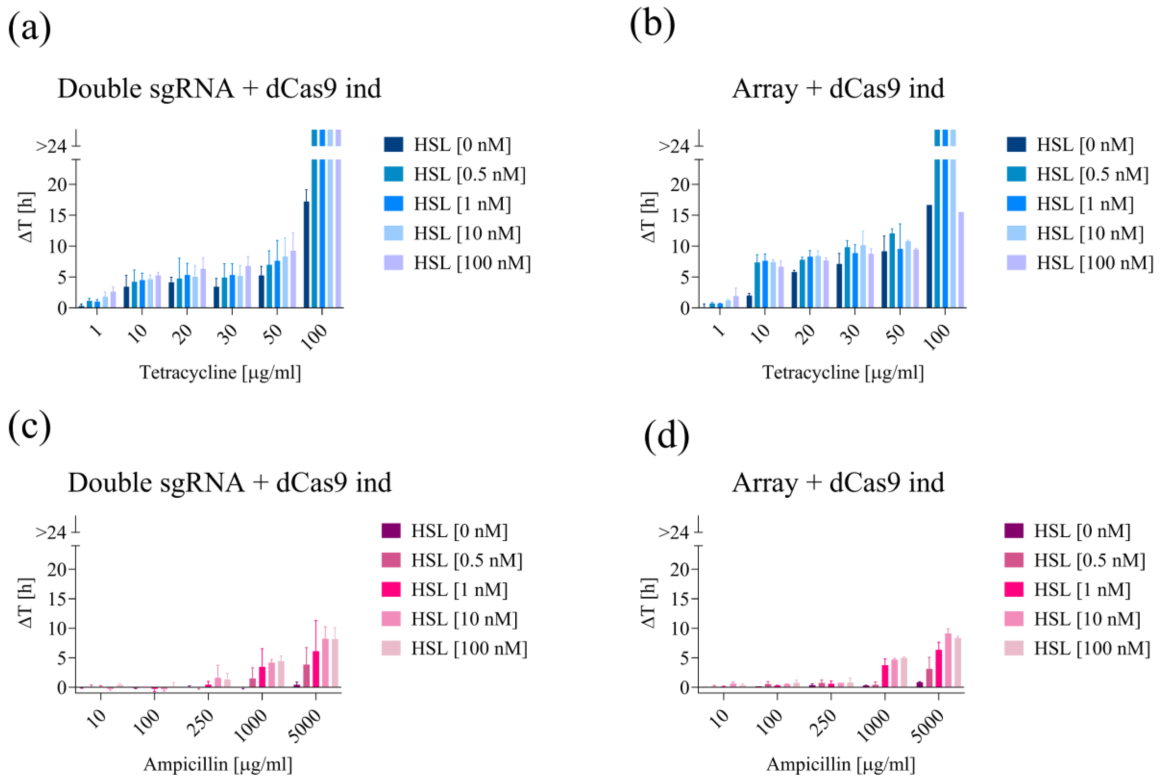

Figure S9.

Growth delays of laboratory strains bearing a one-plasmid CRISPRi platform, including an inducible dCas9 and a two guide RNAs architecture (double sgRNA cassette or CRISPRi array) for different HSL concentrations. The optimal dCas9 expression level for target gene repression was determined by individual TC and AMP growth inhibition assays in which IPTG was added at a fixed concentration to trigger sgRNA or CRISPRi array transcription, and a range of HSL concentrations was used to tune dCas9 level. No growth defect was detected for increasing HSL concentrations in the absence of TET or AMP, confirming the non-toxicity of dCas9 expression in the tested range. **(a-d)** Growth delays (Δt) of tetracycline (a-b) and ampicillin (c-d) *sCRISPRi* strains are shown for the indicated strains relative to the growth profile without antibiotics. *sCRISPRi* strains are engineered with a one-plasmid CRISPRi platform, including HSL-inducible dCas9 and IPTG-inducible double sgRNA cassette (a,c) or CRISPRi array (b,d) transcribing gtetA and $gbla_{promoter}$ simultaneously. Bars represent the mean of at least two independent replicates with error bars indicating standard deviations. The bars over the interrupted axis indicate the antibiotic concentration (MIC) for which cell growth is maintained lower than OD_{600} 0.1 over 24 h. All the data come from experiments in LB media in the presence of IPTG and the indicated HSL concentrations. AT-cr_{sgAT} and AT-cr_{arAT} were used as *sCRISPRi* strains with double-sgRNA and CRISPRi array, respectively, to investigate tetracycline and ampicillin re-sensitization. The 10 nM HSL concentration was selected due to its overall superior performance in terms of growth delay. The comparison between CRISPRi array and double sgRNA cassette showed that the array architecture led to a statistically significant increase (20 to 30%) of delay value ($p<0.05$, ANOVA) for sub-lethal antibiotic levels (AMP=5000 μg/ml and TC>10 μg/ml). As expected from the MIC data above, this analysis also showed that delays were significantly higher for the inducible dCas9 condition than the two-plasmid CRISPRi platform with constitutive dCas9, considering AMP>100 μg/ml ($p<0.05$, ANOVA).

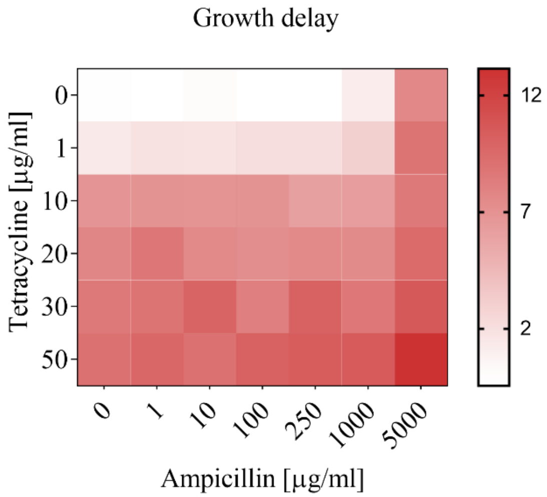

Figure S10.

Growth delays of the laboratory strain bearing a one-plasmid CRISPRi platform including inducible dCas9 and CRISPRi array targeting tetracycline and ampicillin resistance genes, in a combined treatment with the two antibiotics. TC and AMP were administered simultaneously to the TC- and AMP-*sCRISPRi* strain in microplate assays. This test aimed at verifying whether improvements in ampicillin re-sensitization could be achieved by providing a second antibiotic, tetracycline in this test, at concentrations below its individual MIC. The heatmap shows the average growth delay values (Δt) from three independent microplate reader experiments with tetracycline and ampicillin at the indicated concentrations, relative to the growth profile without antibiotics. All the data come from experiments with the same strain, media and condition as in Figure 2c, d. The administration of two antibiotics allowed to increase the recovery of treated cells by 70% compared with the individual treatment with AMP, although regrowth could not be prevented.

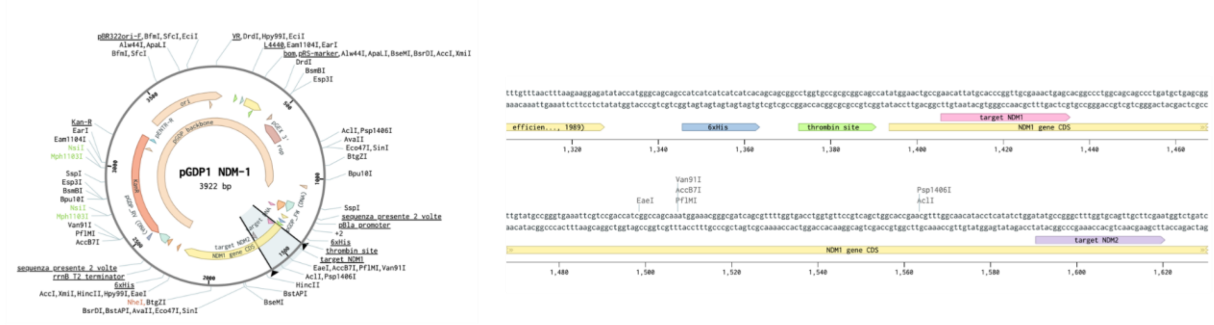

Figure S11.

Target sites of the designed gNDM1 and gNDM2 guides in the *bla*$_{\mathbf{NDM\text{-}1}}$ coding sequence. The codon-optimized *bla*$_{NDM\text{-}1}$ sequence from pGDP1-NDM1 is shown from the Benchling tool. The 20-bp target regions used to design the gNDM1 and gNDM2 spacers are highlighted. The two target sites have a 12- and 197-bp distance from the start codon, respectively.

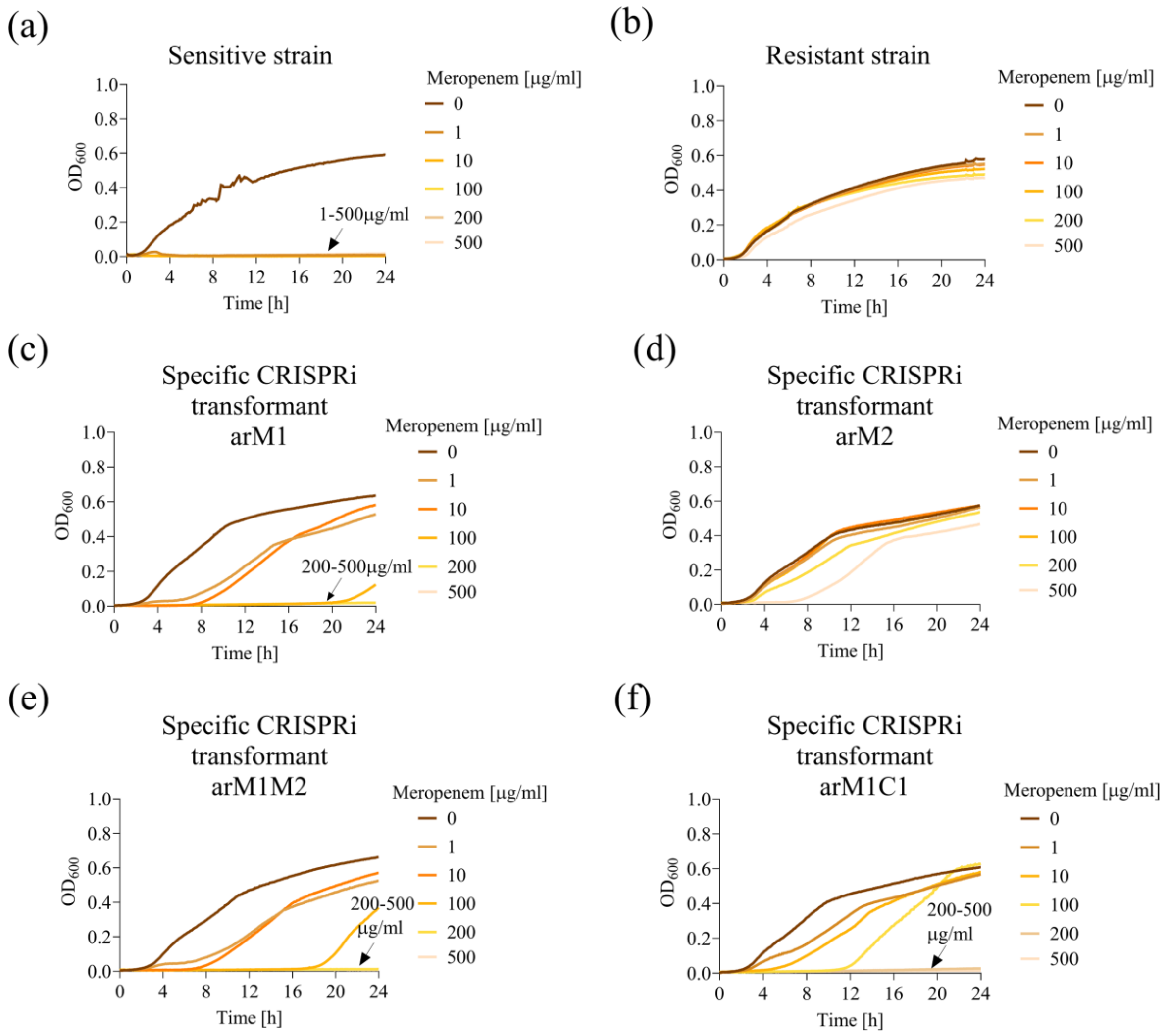

Figure S12.

Re-sensitization to meropenem in laboratory strains (TOP10 F' host) using a one-plasmid CRISPRi platform, including HSL-inducible dCas9 and one of four different CRISPRi arrays targeting *bla*$_{NDM-1}$. (a-f) Growth profiles of *sensitive* (a), *resistant* (b) and *specific CRISPRi (sCRISPRi)* (c-f) strains from microplate assays to investigate meropenem re-sensitization. *sCRISPRi* strains are engineered with a one-plasmid CRISPRi platform, including HSL-inducible dCas9 and IPTG-inducible CRISPRi array transcribing single gNDM1/gNDM2 (c, d), double gNDM1-gNDM2 (e) and double gNDM1-gMCR1 (f) gRNAs. Antibiotic concentrations are provided in the legend. The overlapped curves in which a complete growth inhibition was achieved and the list of the corresponding antibiotic concentrations are indicated with an arrow. All the growth profiles are represented as optical density at 600 nm (OD_{600}) over time. Representative growth curves are shown from a set of at least three independent experiments. All the data come from experiments in LB media in the presence of IPTG and HSL. TOP10F', M-res and M-cr$_{arM1}$/M-cr$_{arM2}$/ M-cr$_{arM1M2}$/M-cr$_{arM1C1}$ were used as *sensitive*, *resistant* and *sCRISPRi* strains, respectively, to investigate meropenem re-sensitization.

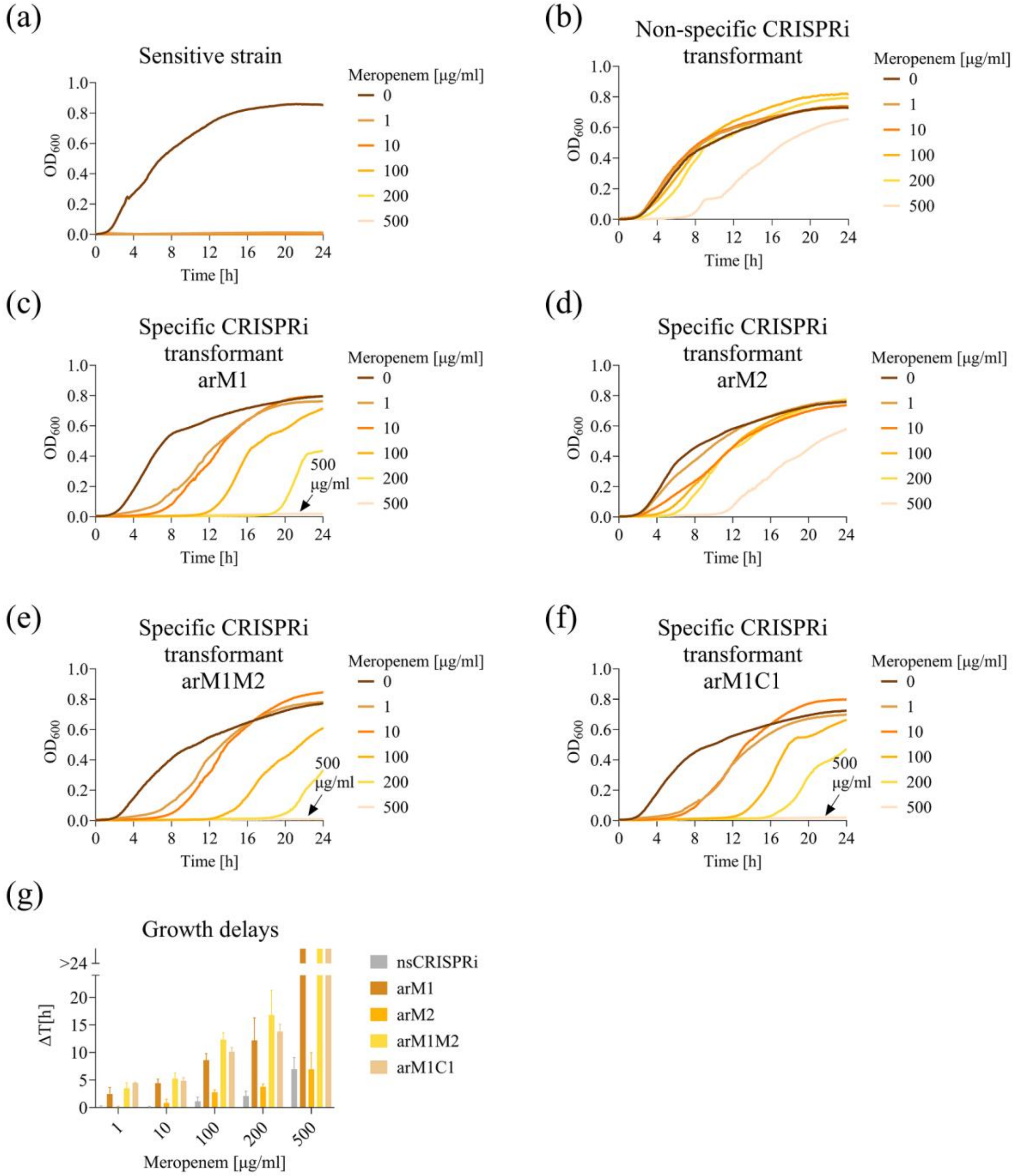

Figure S13.

Re-sensitization to meropenem in K-12 strains (MG1655 host) using a one-plasmid CRISPRi platform, including HSL-inducible dCas9 and one of four different CRISPRi arrays targeting *bla*$_{NDM-1}$. (a-f) Growth profiles of *sensitive* (a), *non-specific CRISPRi (nsCRISPRi)* (b) and *specific CRISPRi (sCRISPRi)* (c-f) strains from microplate assays to investigate meropenem re-sensitization. MG1655 has been used as host to construct one *nsCRISPRi* control (b) and four *sCRISPRi* (c-f) strains. All the data come from experiments with the same media and condition as in Figure S12. Representative growth curves are shown from a set of at least three independent experiments. **(g)** Growth delays (Δt) of the treated strains relative to the growth profile without antibiotics for the *nsCRISPRi* and four *sCRISPRi* strains, corresponding to the guide RNA designs of Figure 3a. Bars represent means and standard deviations (N=3). The bars over the interrupted axis indicate the antibiotic concentration (MIC) for which OD_{600} was lower than 0.1. All the data come from experiments in LB media in the presence of IPTG and HSL. MG1655, MG_M-cr$_{arX14}$ and MG_M-cr$_{arM1}$/MG_M-cr$_{arM2}$/MG_M-cr$_{arM1M2}$/ MG_M-cr$_{arM1C1}$ were used as *sensitive*, *nsCRISPRi* and *sCRISPRi* strains, respectively, to investigate meropenem re-sensitization.

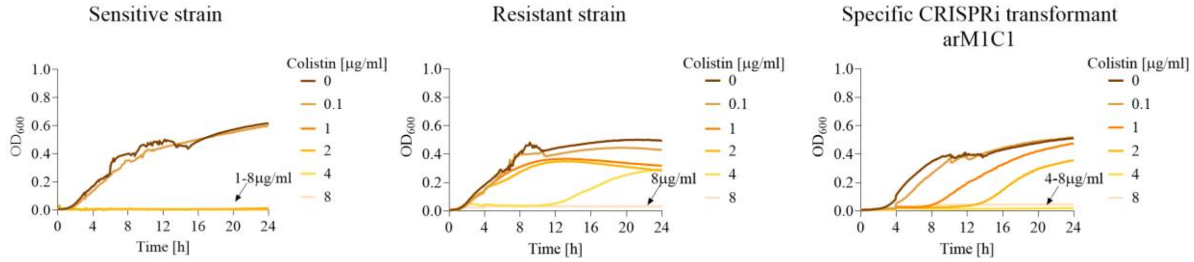

Figure S14.

Re-sensitization to colistin in laboratory strains (TOP10 F' host) using a one-plasmid CRISPRi platform, including HSL-inducible dCas9 and CRISPRi array targeting *mcr-1*. (a-c) Growth profiles of *sensitive* (a), *resistant* (b) and *specific CRISPRi (sCRISPRi)* (c) strains from microplate assays to investigate colistin re-sensitization. *sCRISPRi* strain is engineered with a one-plasmid CRISPRi platform, including HSL-inducible dCas9 and IPTG-inducible CRISPRi array transcribing gMCR1. Antibiotic concentrations are provided in the legend. The overlapped curves in which a complete growth inhibition was achieved and the list of the corresponding antibiotic concentrations are indicated with an arrow. All the growth profiles are represented as optical density at 600 nm (OD_{600}) over time. All the data come from experiments in LB media in the presence of IPTG and HSL. TOP10F', C-res and C-cr_{arM1C1} were used as *sensitive*, *resistant* and *sCRISPRi* strains, respectively, to investigate colistin re-sensitization.

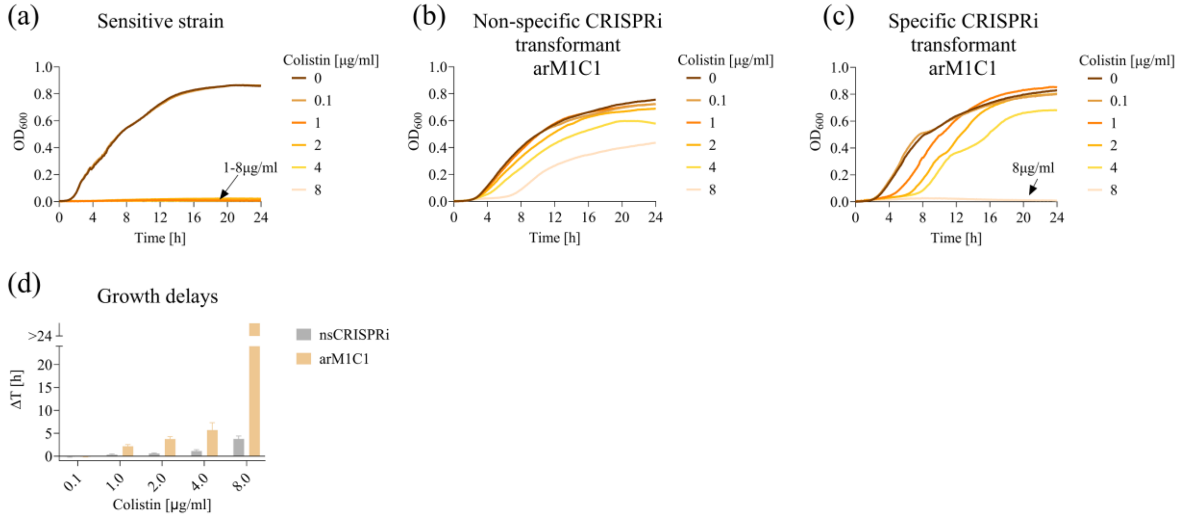

Figure S15.

Re-sensitization to colistin in K-12 strains (MG1655 host) using a one-plasmid CRISPRi platform, including HSL-inducible dCas9 and CRISPRi array targeting *mcr-1*. (a-c) Growth profiles of *sensitive* (a), *non-specific CRISPRi (nsCRISPRi)* (b) and *specific CRISPRi (sCRISPRi)* (c) strains from microplate assays to investigate colistin re-sensitization. MG1655 has been used as a host to construct the *nsCRISPRi* control and *sCRISPRi* strains. All the data come from experiments in the same conditions as in Figure S14. Representative growth curves are shown from a set of at least three independent experiments. **(d)** Growth delays (Δt) of the treated strains relative to the growth profile without antibiotics for the *nsCRISPRi* and *sCRISPRi* strains, corresponding to the gNDM1-gMCR1 guide RNA design (see Figure 3a). Bars represent means and standard deviations (N=3). The bars over the interrupted axis indicate the antibiotic concentration (MIC) for which OD_{600} was lower than 0.1. All the data come from experiments in LB media in the presence of IPTG and HSL. MG1655, MG_C-cr_{crAT} and MG_C-cr_{arM1C1} were used as *sensitive*, *nsCRISPRi* and *sCRISPRi* strains, respectively, to investigate colistin re-sensitization.

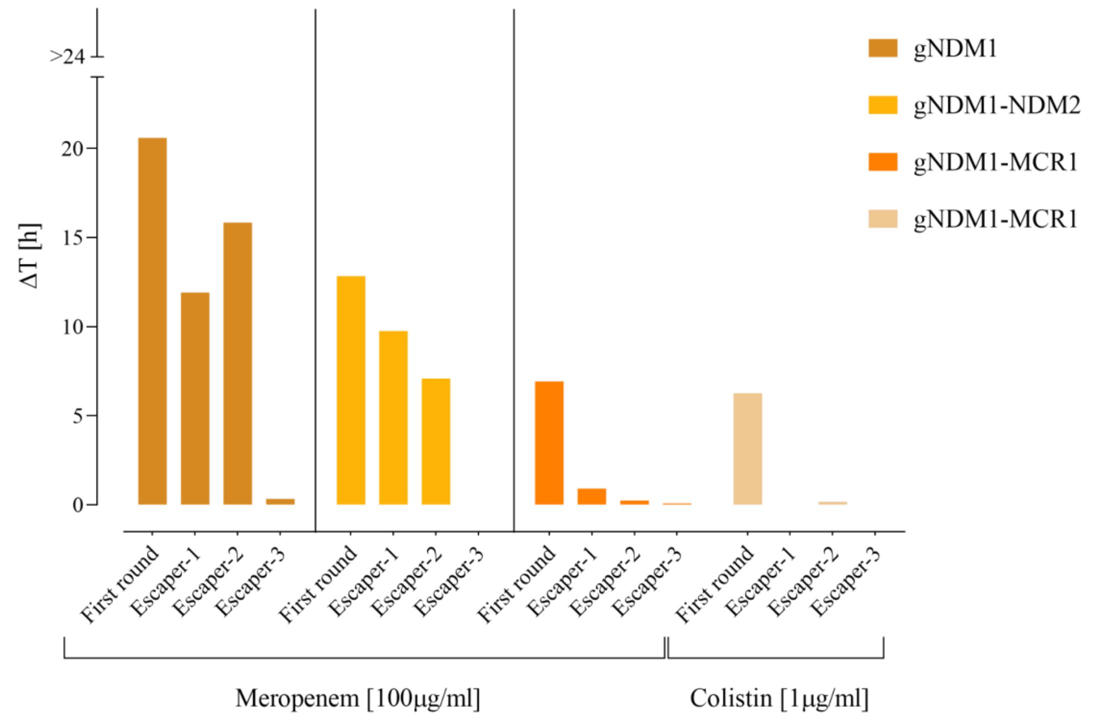

Figure S16.

Growth delays of escaper mutants during second-round experiments. The order of strains is consistent with Figure 3 in the main text. The delays of the first-round experiments are shown for comparison. All the data come from experiments in LB media in the presence of IPTG and HSL.

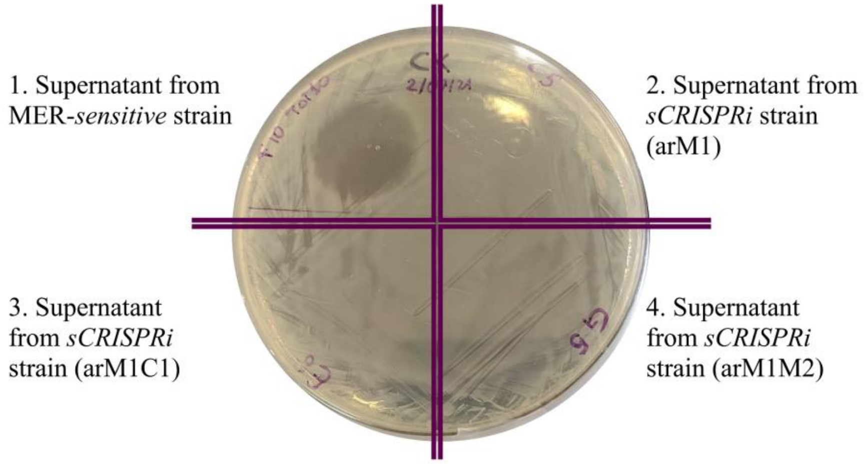

Figure S17.

Supernatant assay with *bla*$_{NDM-1}$-expressing laboratory strains. Supernatants (3 µl) of 24 h lab strain cultures from a meropenem microplate assay were spotted on an LB agar plate in which MER-*sensitive* bacteria were plated. The supernatants were from four strains assayed in presence of 100 µg/ml MER: a *sensitive* strain and three *sCRISPRi* strains (M-cr$_{arM1}$, M-cr$_{arM1M2}$ and M-cr$_{arM1C1}$) in which the growth of escapers was observed.

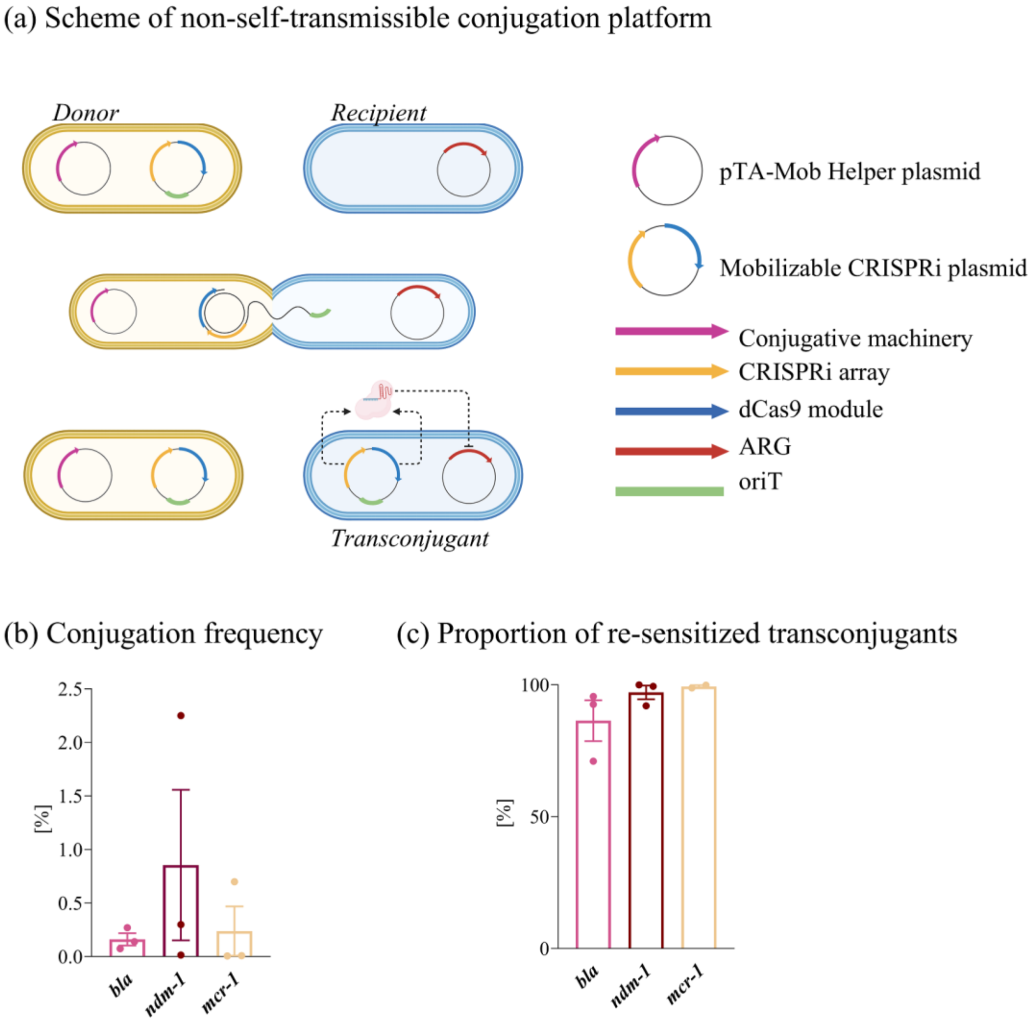

Figure S18.

Horizontal transfer of the CRISPRi plasmid from a donor strain to antibiotic-resistant laboratory strains. (a) Illustration of conjugative transfer, showing the involved plasmids and the resulting plasmid configuration in transconjugant strains. Conjugations were performed using donor cells engineered with a CRISPRi array targeting *bla*$_{\text{TEM-116}}$, *bla*$_{\text{NDM-1}}$, or *mcr-1* gene (strain Donor$_{\text{arAT}}$, Donor$_{\text{arM1}}$, Donor$_{\text{arM1C1}}$), and A-res, M-res, C-res as recipient cells. **(b)** Conjugation efficiency in the three indicated antibiotic-resistant strains. **(c)** Proportion of re-sensitized transconjugant population in the three indicated antibiotic-resistant strains. For conjugations with the A-res recipient strain, expressing no auxiliary antibiotic resistance in the ARG-bearing plasmid, we measured the concentration of transconjugants (CHL + AMP) and survivors (CHL + AMP + IPTG/HSL) to ampicillin administration when the CRISPRi circuitry was induced. For conjugations with M- and C-res recipient strains, we took advantage from the presence of a kanamycin resistance cassette (auxiliary antibiotic resistance in the same ARG-bearing plasmid) to discriminate between transconjugants (CHL + KAN) and survivors (CHL + MER/COL + IPTG/HSL) to meropenem or colistin administration in the presence of the same inducers. In panels (b-c), bars indicate the mean of the represented data points (circles), and error bars represent their standard deviations.

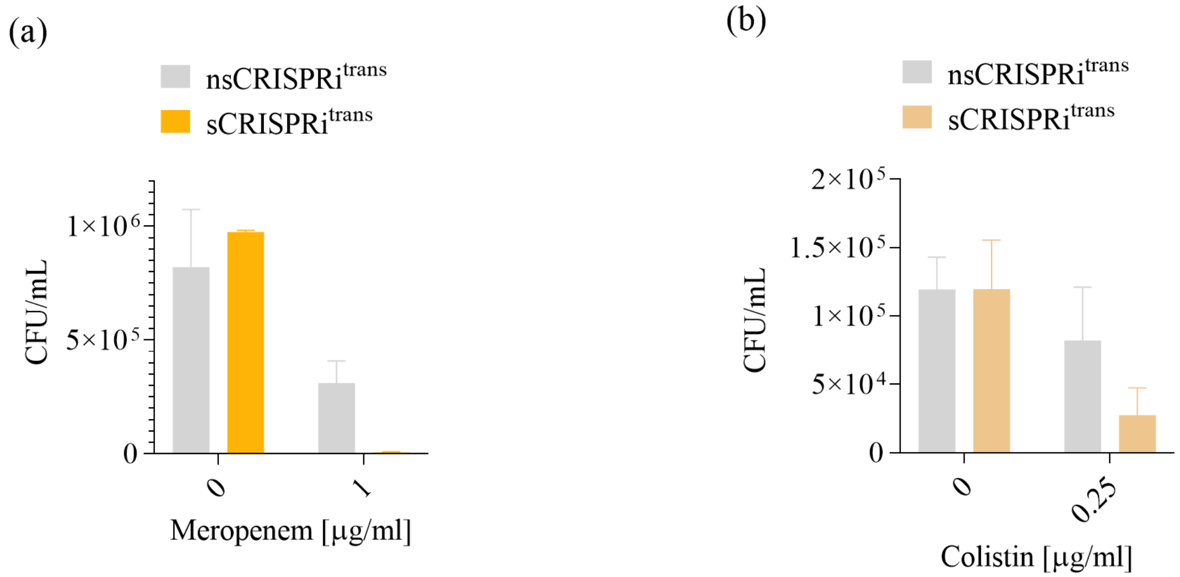

Figure S19.

Meropenem and colistin re-sensitization in agar plate assays for two clinical isolates with *bla*$_{NDM-5}$ and *mcr-1*. (a-b) Colony forming units (CFUs) are shown for the non-specific (*nsCRISPRi^{trans}*) and specific (*sCRISPRi^{trans}*) CRISPRi strains bearing *bla$_{NDM-5}$* (a) or *mcr-1* (b) target gene, after conjugations in which the transconjugants were selected in MH agar in the absence and presence of the indicated antibiotic. The concentration of transconjugants (CHL + LEV/CIP, see Methods section for details on transconjugant selection) and survivors (CHL + MER/COL + IPTG/HSL) was measured in response to meropenem or colistin administration. Bars represent the average value of two (*ndm-5* strains) or three (*mcr-1* strains) independent experiments, with error bars representing standard deviations. M-cr$^{CI}_{arAT}$ and M-cr$^{CI}_{arM(w)}$ were used as *nsCRISPRi^{trans}* and *sCRISPRi^{trans}*, respectively, to investigate meropenem re-sensitization. C-cr$^{CI}_{arAT}$ and C-cr$^{CI}_{arM1C1}$ were used as *nsCRISPRi^{trans}* and *sCRISPRi^{trans}*, respectively, to investigate colistin re-sensitization. Data confirmed the impact of CRISPRi silencing on the expression of both target genes since the concentration of surviving transconjugants decreased up to 163- and 4-fold for MER 1 μg/ml and COL 0.25 μg/ml, respectively. For both antibiotics, in treated strains the colony counts were significantly lower for specific CRISPRi than non-specific controls ($p<0.05$, t-test). Conversely, strains without antibiotics had a comparable number of viable cells varying less than 1.2-fold with no statistical difference.

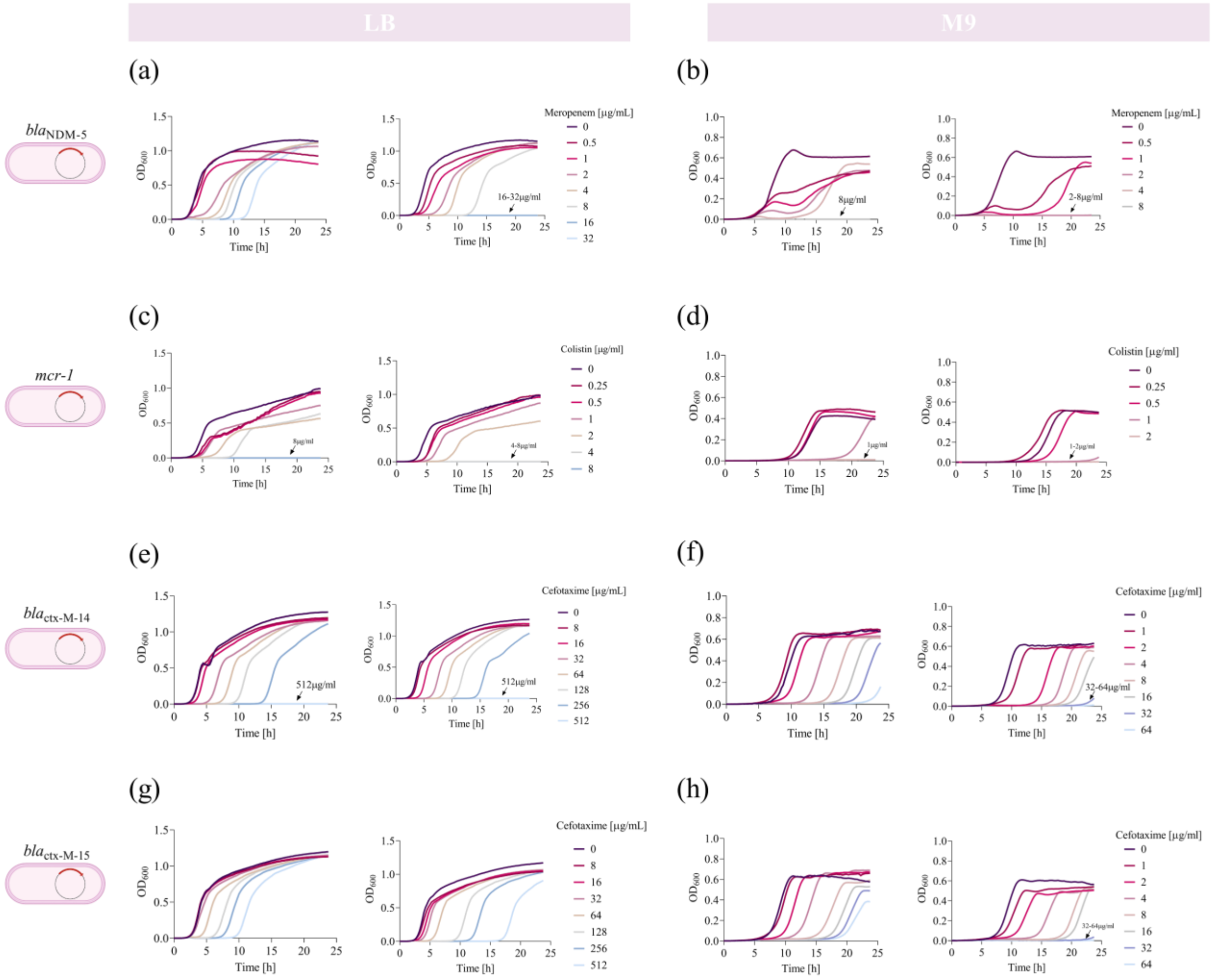

Figure S20.

Antibiotic re-sensitization of four *E. coli* clinical isolates grown in LB and M9 media. (a-h) Growth profiles and delays of *nsCRISPRi^{trans}* (subpanel on the left) and *sCRISPRi^{trans}* (subpanels on the right) from microplate assays to investigate meropenem (a-b), colistin (c-d) and cefotaxime (e-h) re-sensitization. *sCRISPRi^{trans}* are engineered with the one-plasmid CRISPRi platform described in Figure 4. Data in panels (a,c,e,g) were obtained from LB medium microplate assays, and data in panels (b,d,f,h) were obtained from minimal M9 microplate assays. Representative growth curves are shown from a set of at least three independent experiments. Data come from experiments in LB medium in the presence of IPTG and HSL, or M9 medium with lactose in the presence of HSL. Strains used to investigate meropenem, colistin and cefotaxime re-sensitization are listed in the caption of Figure 4.

Supplementary Tables

Table S1.

List of strains used in this work. LC, MC and HC (pSB4C5, pSB3K3 and pSB1A2) vector backbones carrying a collection of synthetic constructs (also listed in Table S2) were inserted in the indicated host strains (TOP10, TOP10F', MG1655, DH10B and clinical isolates). Endogenous plasmids were already present (*E. coli* clinical isolates) or previously incorporated (pTA-Mob, F') in the host strains. The CRISPRi targeted gene is reported, along with a description of the designed function for each strain used in this work. In the "description" section, the antibiotic resistances used as auxiliary markers for transconjugant selection are underlined, while those used as CRISPRi targets are written in bold type. NA=not available. The CI superscript indicates clinical isolates, which are reported in the colored cells of the table.

**Strain**	**Host**	**LC**	**MC**	**HC**	**Endogenous plasmids**	**Target**	**Description**
TOP10	TOP10	-	-	-	-	-	TC- susceptible control
TOP10 F'	TOP10 F'	-	-	-	F' episome	-	TC- resistant control; AMP-, MER-, COL- susceptible control
T-cr$_{sgA_const}$	TOP10 F'	gbla$_{CDS}$	J116dCas9	-	F' episome	-	TC-non-specific CRISPRi transformant with non-targeting gRNA
T-cr$_{sgT_const}$	TOP10 F'	gtetA	J116dCas9	-	F' episome	*tetA*	TC-specific CRISPRi transformant with gtetA targeting tetA CDS
A-res	TOP10 F'	-	-	I13521	F' episome	-	AMP-resistant control and recipient for conjugation with Donor$_{arAT}$; **AMP^{R}**
A-cr$_{sgT_const}$	TOP10 F'	gtetA	J116dCas9	I13521	F' episome	-	AMP-non-specific CRISPRi transformant with non-targeting gRNA
A-cr$_{sgA_const}$	TOP10 F'	gbla$_{promoter}$	J116dCas9	I13521	F' episome	*bla*$_{TEM-116}$	AMP-specific CRISPRi transformant with gbla$_{promoter}$ targeting P$_{bla}$ promoter
A-cr$_{sgA(CDS)_const}$	TOP10 F'	gbla$_{CDS}$	J116dCas9	I13521	F' episome	*bla*$_{TEM-116}$	AMP-specific CRISPRi transformant with gbla$_{CDS}$ targeting *bla*$_{TEM-116}$ CDS
AT-cr$_{sgAT_const}$	TOP10 F'	gbla-gtetA$_{cassette}$	J116dCas9	I13521	F' episome	*bla*$_{TEM-116}$/*tetA*	AMP- and TC- specific CRISPRi transformant with gbla$_{promoter}$ targeting P$_{bla}$ promoter and gtetA targeting *tetA* gene

AT-cr$_{arAT_const}$	TOP10 F'	gbla-gtetA$_{array}$	J116dCas9	I13521	F' episome	*bla*$_{TEM-116}$/*tetA*	AMP- and TC- specific CRISPRi transformant with gbla$_{promoter}$ targeting P$_{bla}$ promoter and gtetA targeting *tetA* gene
AT-cr$_{sgAT}$	TOP10 F'	gbla-gtetA$_{cassette}$ + dCas9	-	I13521	F' episome	*bla*$_{TEM-116}$/*tetA*	AMP- and TC- specific CRISPRi transformant with gbla$_{promoter}$ targeting P$_{bla}$ promoter and gtetA targeting *tetA* gene
AT-cr$_{arAT}$	TOP10 F'	gbla-gtetA$_{array}$ + dCas9	-	I13521	F' episome	*bla*$_{TEM-116}$/*tetA*	AMP- and TC- specific CRISPRi transformant with gbla$_{promoter}$ targeting P$_{bla}$ promoter and gtetA targeting *tetA* gene
M-res	TOP10 F'	-	pGDP1 NDM-1	-	F' episome	-	MER-resistant control and recipient for conjugation with Donor$_{arM1}$; KAN^{R}, **MER^{R}**
M-cr$_{arM1}$	TOP10 F'	oriT_gNDM1$_{array}$ + dCas9	pGDP1 NDM-1	-	F' episome	*bla*$_{NDM-1}$	MER-specific CRISPRi transformant with gNDM1 targeting *bla*$_{NDM-1}$ CDS at site 1
M-cr$_{arM2}$	TOP10 F'	oriT_gNDM2$_{array}$ + dCas9	pGDP1 NDM-1	-	F' episome	*bla*$_{NDM-1}$	MER-specific CRISPRi transformant with gNDM2 targeting *bla*$_{NDM-1}$ CDS at site 2
M-cr$_{arM1M2}$	TOP10 F'	oriT_gNDM1-gNDM2$_{array}$ + dCas9	pGDP1 NDM-1	-	F' episome	*bla*$_{NDM-1}$	MER-specific CRISPRi transformant with gNDM1 and gNDM2 targeting *bla*$_{NDM-1}$ CDS at site 1 and site 2
M-cr$_{arM1C1}$	TOP10 F'	oriT_gNDM1-gMCR1$_{array}$ + dCas9	pGDP1 NDM-1	-	F' episome	*bla*$_{NDM-1}$	MER-specific CRISPRi transformant with gNDM1 targeting *bla*$_{NDM-1}$ CDS at site 1
C-res	TOP10 F'	-	pGDP2 MCR-1	-	F' episome	-	COL-resistant control and recipient for conjugation with Donor$_{arM1C1;}$ KAN^{R}, **COL^{R}**

C-cr$_{arM1C1}$	TOP10 F'	gNDM1-gMCR1$_{array}$ + dCas9	pGDP2 MCR-1	-	F' episome	*mcr-1*	COL-specific CRISPRi transformant with gMCR1 targeting *mcr-1* CDS
MG1655	MG1655	-	-	-	-	-	Model K-12 host; MER- and COL-susceptible control
MG_M-cr$_{arX14}$	MG1655	oriT_gCTX-M-14 + dCas9	pGDP1 NDM-1	-	-	-	Model K-12 host; MER-non-specific CRISPRi transformant with non-targeting gRNA
MG_M-cr$_{arM1}$	MG1655	oriT_gNDM1$_{array}$ + dCas9	pGDP1 NDM-1	-	-	*bla*$_{NDM-1}$	Model K-12 host; MER-specific CRISPRi transformant with gNDM1 targeting *bla*$_{NDM-1}$ CDS at site 1
MG_M-cr$_{arM2}$	MG1655	oriT_gNDM2$_{array}$ + dCas9	pGDP1 NDM-1	-	-	*bla*$_{NDM-1}$	Model K-12 host; MER-specific CRISPRi transformant with gNDM2 targeting *bla*$_{NDM-1}$ CDS at site 2
MG_M-cr$_{arM1M2}$	MG1655	oriT_gNDM1-gNDM2$_{array}$ + dCas9	pGDP1 NDM-1	-	-	*bla*$_{NDM-1}$	Model K-12 host; MER-specific CRISPRi transformant with gNDM1 and gNDM2 targeting *bla*$_{NDM-1}$ CDS at site 1 and site 2
MG_M-cr$_{arM1C1}$	MG1655	oriT_gNDM1-gMCR1$_{array}$ + dCas9	pGDP1 NDM-1	-	-	*bla*$_{NDM-1}$	Model K-12 host; MER-specific CRISPRi transformant with gNDM1 targeting *bla*$_{NDM-1}$ CDS at site 1
MG_C-cr$_{crAT}$	MG1655	gbla-gtetA$_{array}$ + dCas9	pGDP2 MCR-1	-	-	-	Model K-12 host; COL-non-specific CRISPRi transformant with non-targeting gRNA
MG_C-cr$_{arM1C1}$	MG1655	gNDM1-gMCR1$_{array}$ + dCas9	pGDP2 MCR-1	-	-	*mcr-1*	Model K-12 host; COL-specific CRISPRi transformant with gMCR1 targeting *mcr-1* CDS

Donor$_{arAT}$	DH10B	oriT_gAMP-gtetA$_{array}$ + dCas9	-	-	p TA-Mob	-	Donor for conjugation with A-res, M-res^{CI}, C-res^{CI}
Donor$_{arM1}$	DH10B	oriT_gNDM1$_{array}$ + dCas9	-	-	p TA-Mob	-	Donor for conjugation with M-res
Donor$_{arM1C1}$	DH10B	oriT_gNDM1-gMCR1$_{array}$ + dCas9	-	-	p TA-Mob	-	Donor for conjugation with C-res and C-res^{CI}
A-cr$_{arAT}$ ^{trans}	TOP10 F'	oriT_gbla-gtetA$_{array}$ + dCas9	-	I13521	-	*bla*$_{TEM-116}$	AMP-specific CRISPRi transconjugant with with gbla$_{promoter}$ targeting P$_{bla}$ promoter
M-cr$_{arM1}$^{trans}	TOP10 F'	oriT_gNDM1$_{array}$ + dCas9	pGDP1 NDM-1	-	-	*bla*$_{NDM-1}$	MER-specific CRISPRi transconjugant with gNDM1 targeting *bla*$_{NDM-1}$ CDS at site 1
C-cr$_{arM1C1}$^{trans}	TOP10 F'	oriT_gNDM1-gMCR$_{array}$ + dCas9	pGDP2 MCR-1	-	-	*mcr-1*	COL-specific CRISPRi transconjugant with gMCR1 targeting *mcr-1* CDS
Donor$_{arM(wt)}$	DH10B	oriT_gNDM(wt) + dCas9	-	-	p TA-Mob	-	Donor for conjugation with M-res^{CI}, X14-res^{CI}, X15-res^{CI}
Donor$_{arX14}$	DH10B	oriT_gCTX-M-14 + dCas9	-	-	p TA-Mob	-	Donor for conjugation with X14-res^{CI}
Donor$_{arX15}$	DH10B	oriT_gCTX-M-15 + dCas9	-	-	p TA-Mob	-	Donor for conjugation with X15-res^{CI}
M-res^{CI}	ST167	-	-	-	*bla*$_{NDM-5}$ bearing plasmid	-	MER-resistant recipient for conjugation with Donor$_{arM(wt);}$ LEV^{R}, **MER^{R}**

M-cr$^{CI}_{arAT}$	ST167	oriT_gbla-gtetA$_{array}$ + dCas9	-	-	*bla*$_{NDM-5}$ bearing plasmid	-	MER (wt)-non-specific CRISPRi transconjugant with non-targeting gRNA
M-cr$^{CI}_{arM(wt)}$	ST167	oriT_gNDM(wt) + dCas9	-	-	*bla*$_{NDM-5}$ bearing plasmid	*bla*$_{NDM-5}$	MER (wt)-specific CRISPRi transconjugant with gNDM(wt) targeting *bla*$_{NDM-5}$ CDS
C-res^{CI}	ST617	-	-	-	-	-	Recipient for conjugation with Donor$_{arM1C1}$, <u>CIP^{R}</u>, **COL^{R}**
C-cr$^{CI}_{arAT}$	ST617	oriT_gbla-gtetA$_{array}$ + dCas9	-	-	-	-	COL (wt)-non-specific CRISPRi transconjugant with non-targeting gRNA
C-cr$^{CI}_{arM1C1}$	ST617	oriT_gNDM1-gMCR1$_{array}$ + dCas9	-	-	-	*mcr-1*	COL (wt)-specific CRISPRi transconjugant with gMCR1 targeting *mcr-1* CDS
X14-res^{CI}	ST69	-	-	-	-	-	Recipient for conjugation with Donor$_{arX14}$
X14-cr$^{CI}_{arM(wt)}$	ST69	oriT_gNDM1$_{array}$ + dCas9	-	-	-	-	CTX-M-14-non-specific CRISPRi transconjugant with non-targeting gRNA
X14-cr$^{CI}_{arX14}$	ST69	oriT_gCTX-M-14 + dCas9	-	-	-	*bla*$_{ctx-M-14}$	CTX-M-14-specific CRISPRi transconjugant with gCTX-M-14 targeting *bla*$_{ctx-M-14}$ CDS
X15-res^{CI}	ST405	-	-	-	-	-	Recipient for conjugation with Donor$_{arX15}$

X15-cr^{CI} $_{arM(wt)}$	ST405	oriT_gNDM1$_{array}$ + dCas9	-	-	-	-	CTX-M-15-non-specific CRISPRi transconjugant with non-targeting gRNA
X15-cr^{CI} $_{arX15}$	ST405	oriT_gCTX-M-15 + dCas9	-	-	-	*bla*$_{ctx\text{-}M\text{-}15}$	CTX-M-15-specific CRISPRi transconjugant with gCTX-M-15 targeting *bla*$_{ctx\text{-}M\text{-}15}$ CDS

Table S2.
List of recombinant plasmids used in this work. The replication origin, the plasmid code and a description of the genetic parts composing each plasmid are reported. Plasmid codes are relative to the MIT Registry of Standard Biological Parts (http://parts.igem.org), in which sequences are available; the BBa_ prefix is omitted. These codes indicate the inserts of the LC, MC and HC (pSB4C5, pSB3K3 and pSB1A2) vector backbones.

**Plasmid**	**Copy Number**	**Replication origin**	**Code**	**Description**
I3521	HC	mutated pMB1	pSB1A2-I13521	Recombinant vector carrying the $bla_{\text{TEM-116}}$ gene under the P_{bla} promoter
J116dCas9	MC	p15A	pSB3K3-J107202	Recombinant vector carrying a constitutive dCas9 cassette under the P_{J23116} promoter
pGDP1-NDM1	MC	pBR322	Addgene n.#112883	Recombinant vector carrying a codon-optimized version of the $bla_{\text{NDM-1}}$ gene under the P_{bla} promoter
pGDP2-MCR1	MC	pBR322	Addgene n.#118404	Recombinant vector carrying *mcr-1* gene under the P_{Llac01} promoter
gtetA	LC	pSC101	pSB4C5-J107161	IPTG-inducible single sgRNA cassette transcribing gtetA under the P_{Llac01} promoter
$gbla_{promoter}$	LC	pSC101	pSB4C5-J107162	IPTG-inducible single sgRNA cassette transcribing $gbla_{promoter}$ under the P_{Llac01} promoter
$gbla_{CDS}$	LC	pSC101	pSB4C5-J107163	IPTG-inducible single sgRNA cassette transcribing $gbla_{CDS}$ under the P_{Llac01} promoter
gbla-$gtetA_{cassette}$	LC	pSC101	pSB4C5-J107164	IPTG-inducible double sgRNA cassette transcribing $gbla_{promoter}$ and gtetA under the P_{Llac01} promoter
gbla-$gtetA_{array}$	LC	pSC101	pSB4C5-J107165	IPTG-inducible CRISPRi array transcribing $gbla_{promoter}$ and gtetA under the P_{Llac01} promoter

gbla-gtetA$_{cassette}$ + dCas9	LC	pSC101	pSB4C5-J107166	IPTG-inducible double sgRNA cassette transcribing gbla$_{promoter}$ and gtetA under the P$_{Llac01}$ promoter + HSL-inducible dCas9 cassette under P$_{lux}$ promoter
gbla-gtetA$_{array}$ + dCas9	LC	pSC101	pSB4C5-J107167	IPTG-inducible CRISPRi array transcribing gbla$_{promoter}$ and gtetA under the P$_{Llac01}$ promoter + HSL-inducible dCas9 cassette under the P$_{lux}$ promoter
oriT_gNDM1$_{array}$ + dCas9	LC	pSC101	pSB4C5-J107168	IPTG-inducible CRISPRi array transcribing gNDM1 under the P$_{Llac01}$ promoter + HSL-inducible dCas9 under the P$_{lux}$ promoter, with the origin of transfer sequence (oriT) from pRK2
oriT_gNDM2$_{array}$ + dCas9	LC	pSC101	pSB4C5-J107169	IPTG-inducible CRISPRi array transcribing gNDM2 under the P$_{Llac01}$ promoter + HSL-inducible dCas9 under the P$_{lux}$ promoter, with the origin of transfer sequence (oriT) from pRK2
oriT_gNDM1-gNDM2$_{array}$ + dCas9	LC	pSC101	pSB4C5-J107170	IPTG-inducible CRISPRi array transcribing gNDM1 and gNDM2 under the P$_{Llac01}$ promoter + HSL-inducible dCas9 under the P$_{lux}$ promoter, with the origin of transfer sequence (oriT) from pRK2
oriT_gNDM1-gMCR1$_{array}$ + dCas9	LC	pSC101	pSB4C5-J107171	IPTG-inducible CRISPRi array transcribing gNDM1 and gMCR1 under the P$_{Llac01}$ promoter + HSL-inducible dCas9 under the P$_{lux}$ promoter, with the origin of transfer sequence (oriT) from pRK2.
oriT_gbla-gtetA$_{array}$ + dCas9	LC	pSC101	pSB4C5-J107172	Same as gbla-gtetA$_{array}$ + dCas9, with the origin of transfer sequence (oriT) from pRK2
oriT_gNDM(wt) + dCas9	LC	pSC101	pSB4C5-J107175	IPTG-inducible CRISPRi array transcribing gNDM$_{(wt)}$ under the P$_{Llac01}$ promoter + HSL-inducible dCas9 under the P$_{lux}$ promoter. gNDM(wt) targets the wt sequence of the *bla*$_{NDM-5}$ gene
oriT_gCTX-M-14 + dCas9	LC	pSC101	pSB4C5-J107176	IPTG-inducible CRISPRi array transcribing gCTX-M-14 under the P$_{Llac01}$ promoter + HSL-inducible dCas9 under the P$_{lux}$ promoter.
oriT_gCTX-M-15 + dCas9	LC	pSC101	pSB4C5-J107177	IPTG-inducible CRISPRi array transcribing gCTX-M-15 under the P$_{Llac01}$ promoter + HSL-inducible dCas9 under the P$_{lux}$ promoter.

Table S3.
List of primers used in this work for escaper analysis. For each template, the primer pairs used for DNA sequencing are reported along with a description of the specific part to be sequenced.

Template	Primer Pair	Sequence (5'-3')	Description
gNDM1 / gNDM1-gNDM2 / gNDM1-gMCR1$_{array}$ + dCas9	C0062_VF	gaatgtttagcgtgggcatg	Sequencing of CRISPRi array and dCas9 gene from the circuits encoding *bla*$_{NDM-1}$ and *mcr-1* spacers
	FW_dCas9	caatccatcactggtctttatg	
pGDP1 NDM-1	FW_pGDP	cgtatcggtgattcattctgc	Sequencing of *bla*$_{NDM-1}$ and *mcr-1* gene
pGDP2 MCR-1	RV_pGDP	gcagtttcatttgatgctcgat	

Table S4.
List of IC_{99} values determined with agar plate assays. All the values are expressed as (µg/mL).

	Sensitive	Resistant	Specific CRISPRi transformant				
**Antibiotic [µg/mL]**			single sgRNA	sgRNA cassette + dCas9 const	Array + dCas9 const	sgRNA cassette + dCas9 ind	Array + dCas9 ind
**TC**	0.5	50	5	/	/	/	5
**AMP**	5	> 1000	50 (gbla$_{promoterr}$) 150 (gbla$_{CDS}$)	/	/	/	200
**MER**	0.0312	10	/	/	/	/	0.125
**COL**	0.0312	2	/	/	/	/	0.125

Table S5.
MIC values of recombinant strains obtained from the TOP10 F' or MG1655 hosts. Values were determined via microplate assays carried out in LB medium. Values are relative to tests in the presence of inducers (IPTG = 500 µM and HSL = 10 nM when required). TC=tetracycline; AMP=ampicillin; MER=meropenem; COL=colistin.

Strain	Specific CRISPRi	Non-specific CRISPRi	Antibiotic	MIC (µg/ml)
TOP10 (TC susceptible)	-	-	TC	10
TOP10F' (TC resistant)	-	-	TC	>100

T-cr$_{sgA_const}$ (TC resistant)	-	non-targeting gRNA	TC	>100
T-cr$_{sgT_const}$	single sgRNA	-	TC	100
AT-cr$_{sgAT_const}$	double sgRNA cassettes + dCas9 const	-	TC	>100
AT-cr$_{arAT_const}$	double-spacer array + dCas9 const	-	TC	>100
AT-cr$_{sgAT}$	double sgRNA cassettes + dCas9 ind	-	TC	100
AT-cr$_{arAT}$	double-spacer array + dCas9 ind	-	TC	100
TOP10F' (AMP susceptible)	-	-	AMP	10
A-res (AMP resistant)	-	-	AMP	>5000
A-cr$_{sgT_const}$ (AMP resistant)	-	non-targeting gRNA	AMP	>5000
A-cr$_{sgA_const}$	single sgRNA	-	AMP	5000
A-cr$_{sgA(CDS)_const}$	single sgRNA	-	AMP	>5000
AT-cr$_{sgAT_const}$	double sgRNA cassettes + dCas9 const	-	AMP	>5000
AT-cr$_{arAT_const}$	double-spacer array + dCas9 const	-	AMP	>5000
AT-cr$_{sgAT}$	double sgRNA cassettes + dCas9 ind	-	AMP	>5000
AT-cr$_{arAT}$	double-spacer array + dCas9 ind	-	AMP	>5000
TOP10F' (MER susceptible)	-	-	MER	<1
M-res (MER resistant)	-	-	MER	>500
M-cr$_{arM1}$	single-spacer array + dCas9 ind	-	MER	200
M-cr$_{arM2}$	single-spacer array + dCas9 ind	-	MER	>500
M-cr$_{arM1M2}$	double-spacer array + dCas9 ind	-	MER	200
M-cr$_{arM1C1}$	double-spacer array + dCas9 ind	-	MER	200
TOP10F' (COL susceptible)	-	-	COL	1
C-res (COL resistant)	-	-	COL	8
C-cr$_{arM1C1}$	double-spacer array + dCas9 ind	-	COL	4
MG1655 (MER susceptible)	-	-	MER	<1
MG_M-cr$_{arX14}$ (MER resistant)	-	non-targeting gRNA	MER	>500
MG_M-cr$_{arM1}$	single-spacer array + dCas9 ind	-	MER	500
MG_M-cr$_{arM2}$	single-spacer array + dCas9 ind	-	MER	>500
MG_M-cr$_{arM1M2}$	double-spacer array + dCas9 ind	-	MER	500
MG_M-cr$_{arM1C1}$	double-spacer array + dCas9 ind	-	MER	500
MG1655 (COL susceptible)	-	-	COL	1
MG_C-cr$_{sgAT}$ (COL resistant)	-	non-targeting gRNA	COL	>8
MG_C-cr$_{arM1C1}$	double-spacer array + dCas9 ind	-	COL	8

Table S6.

MIC values of clinical isolates. Values were determined via microplate assays carried out in the indicated media, or via microdilution assays according to EUCAST guidelines. Values are relative to tests in the presence of inducers (IPTG = 500 µM, when required, and HSL = 10 nM). Antibiotics: MER=meropenem; COL=colistin; CTX=cefotaxime. Growth media: MH=Mueller Hinton broth; M9=minimal M9 medium with lactose as carbon source; HU=human urine medium. MIC and EUCAST MIC refer to the minimum inhibitory concentration determined in microplate assays via dynamic plate reader experiments, and in microdilution assays according to the EUCAST guidelines, respectively. "-" not tested.

**Strain**	**Specific CRISPRi**	**Non-specific CRISPRi**	**Antibiotic**	**MIC in MH (µg/ml)**	**MIC in LB (µg/ml)**	**MIC in M9 (µg/ml)**	**MIC in HU (µg/ml)**	**EUCAST MIC (µg/ml)**
M-cr^{CI}_{arAT} (MER resistant)	-	non-targeting gRNA	MER	>32	>32	8	-	256
M-$cr^{CI}_{arM(wt)}$	single-spacer array	-	MER	8	16	2	-	64
C-cr^{CI}_{arAT} (COL resistant)	-	non-targeting gRNA	COL	8	8	2	-	8
C-cr^{CI}_{arM1C1}	double-spacer array	-	COL	4	4	1	-	4
X14-$cr^{CI}_{arM(wt)}$ (CTX resistant)	-	non-targeting gRNA	CTX	>512	512	>64	-	>512
X14-cr^{CI}_{arX14}	single-spacer array	-	CTX	256	512	32	-	>512
X15-$cr^{CI}_{arM(wt)}$ (CTX resistant)	-	non-targeting gRNA	CTX	>512	>512	>64	>512	>512
X15-cr^{CI}_{arX15}	single-spacer array	-	CTX	>512	>512	32	256	>512
